# Alzheimer’s disease disrupts intra-adipose neurovascular contact

**DOI:** 10.1016/j.jlr.2025.100886

**Published:** 2025-08-25

**Authors:** Michelle Kwong, Jianting Sheng, Li Yang, Stephen T.C. Wong

**Affiliations:** 1 Systems Medicine and Bioengineering Department, Houston Methodist Neal Cancer Center, Houston Methodist Hospital, Houston, TX; 2 Ting Tsung & Wei Fong Chao Center for BRAIN, Houston Methodist Academic Institute and Weill Cornell Medicine, Houston, TX

Alzheimer’s disease (AD) induces autonomic nervous system dysfunction (dysautonomia), which exacerbates cardiovascular and metabolic comorbidities, yet the role of dysautonomia in metabolic dysregulation remains underexplored (1). Using three-dimensional whole-mount imaging, we revealed that AD disrupts organized neurovascular bundles in subcutaneous adipose tissue—critical structures for brain-fat communication via hormonal signaling and sympathetic innervation (2) (Supplemental Fig. S1). In healthy adipose tissue, sympathetic nerves align closely with blood vessels, forming neurovascular bundles (Figs. 1A and Supplemental Fig. S2A and B, Movie S1-S2), whereas in AD, we observed structural decoupling (Figs. 1B and Supplemental Fig. S2C, Movie S3), loss of spatial proximity (Figs. 1C and Supplemental Fig. S2D, Movie S4), and abrogated sympathetic signals (Figs. 1D and Supplemental Fig. S2E and F, Movie S5-S6), implicating impaired neural control of adipose metabolism.

**Fig. 1:**
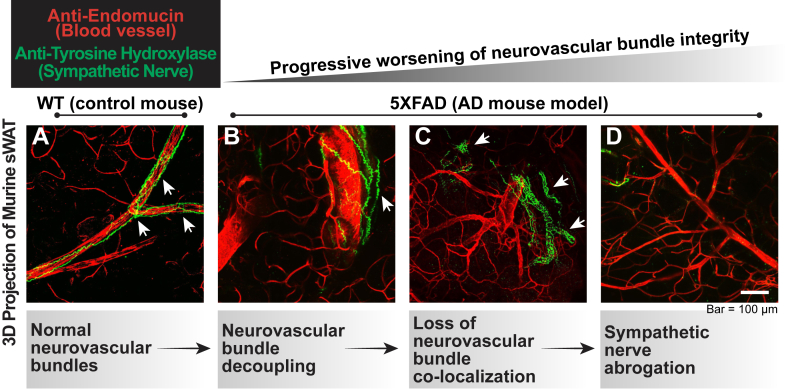
Progressive disruption of neurovascular bundles in subcutaneous adipose tissue of 5XFAD mice.

**Equipment, reagents, and animals:** Whole-mount immunostaining was performed as previously described (3). Imaging was conducted using a 20X objective on an Olympus FV3000 confocal microscope. 5XFAD (MMRRC-#034848-JAX lab) and wild-type mice (3–4 mice/group) at 25 weeks of age were used.

## Supplemental data

This article contains supplemental data.

## Conflict of interest

The authors declare that they have no conflicts of interest with the contents of this article.

## Supplementary data

Movie S6

Supplemental Figure S1: Adipose–brain crosstalk and histological features of sWAT in WT and 5XFAD mice. A. Schematic representation of neurovascular bundles mediating the crosstalk between adipose tissue and the brain. B-E. Representative hematoxylin and eosin staining of sWAT sections from WT (B) and 5XFAD (D) mice. Insets outlined by dotted boxes in B and D are shown at higher magnification in C and E, respectively. Hematoxylin and eosin staining was used to assess adipocyte size, morphology, and lipid droplet characteristics. Both WT and 5XFAD sWAT display heterogeneous adipocyte populations, including large unilocular adipocytes and smaller multilocular-like cells. Scale bars, 50 μm (B & D); 20 μm (C & E).

Supplemental Figure S2: Representative images showing progressive pathological remodeling of neurovascular bundles in the Alzheimer’s disease model.
