# Supplementary figures and images for: Alzheimer’s disease disrupts intra-adipose neurovascular contact
Source: J Lipid Res. 2025 Aug 25;66(10):100886. doi: 10.1016/j.jlr.2025.100886 (PMC12481055; doi:10.1016/j.jlr.2025.100886)

Figure S1

A

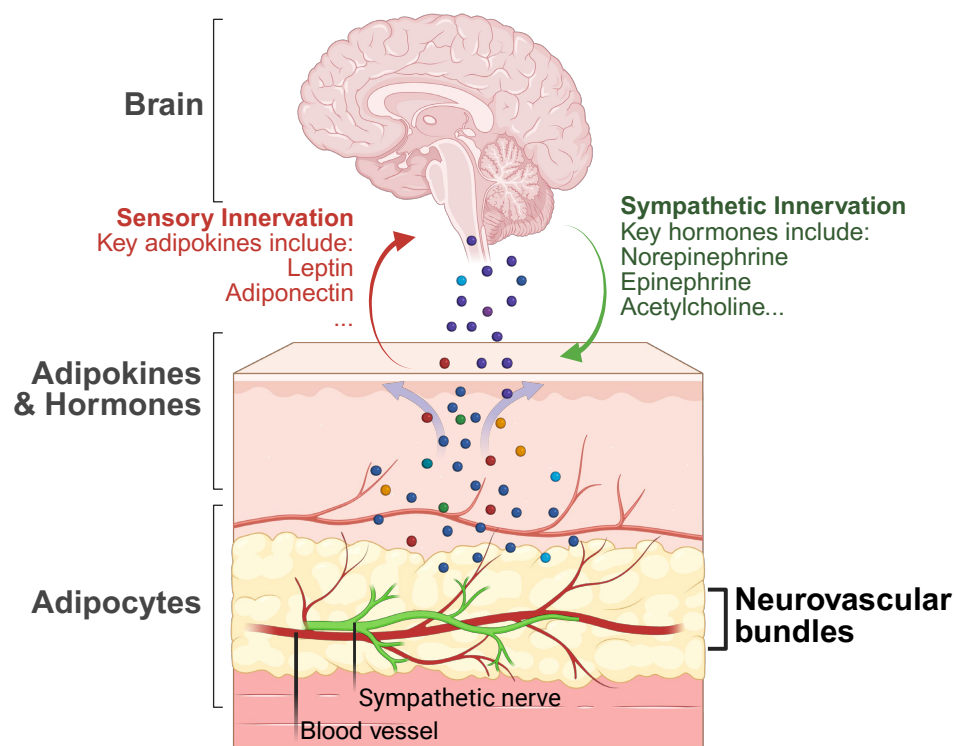

B

WT\_sWAT

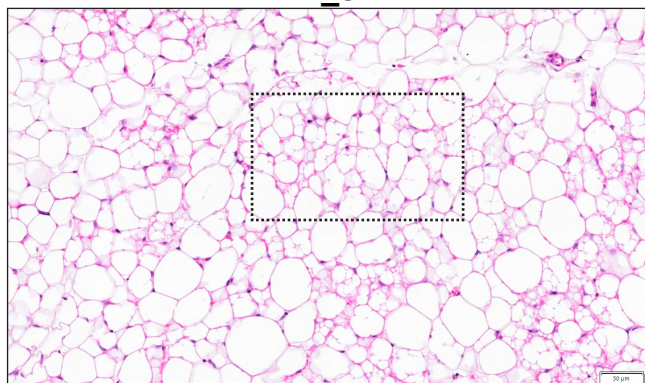

D

5XFAD\_sWAT

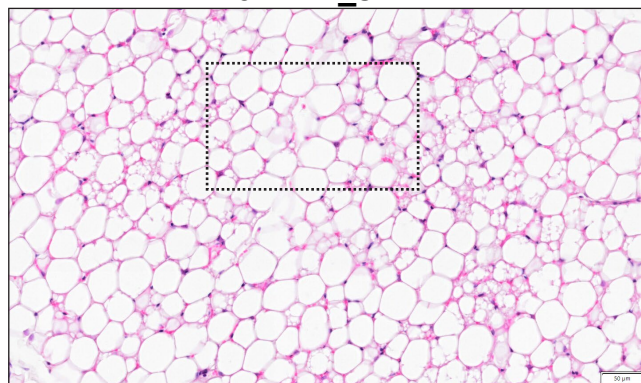

C

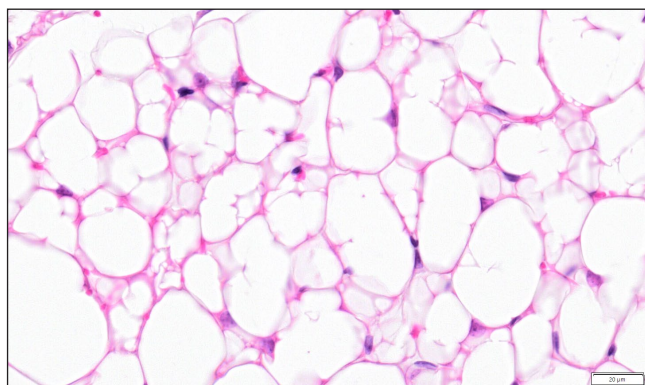

E

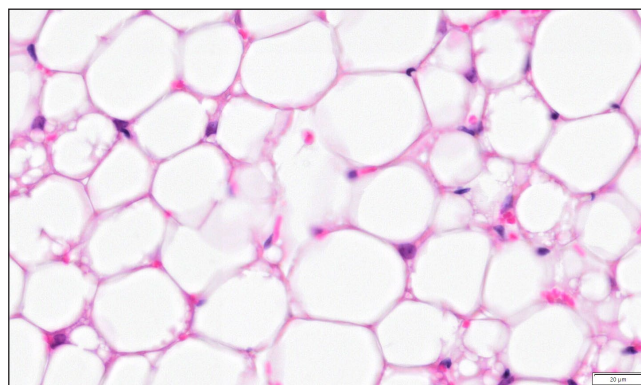

Supplement: Supplemental Figure S1: Adipose–brain crosstalk and histological features of sWAT in WT and 5XFAD mice. A. Schematic representation of neurovascular bundles mediating the crosstalk between adipose tissue and the brain. B-E. Representative hematoxylin and eosin staining of sWAT sections from WT (B) a [file mmc7.pdf]

Figure S2

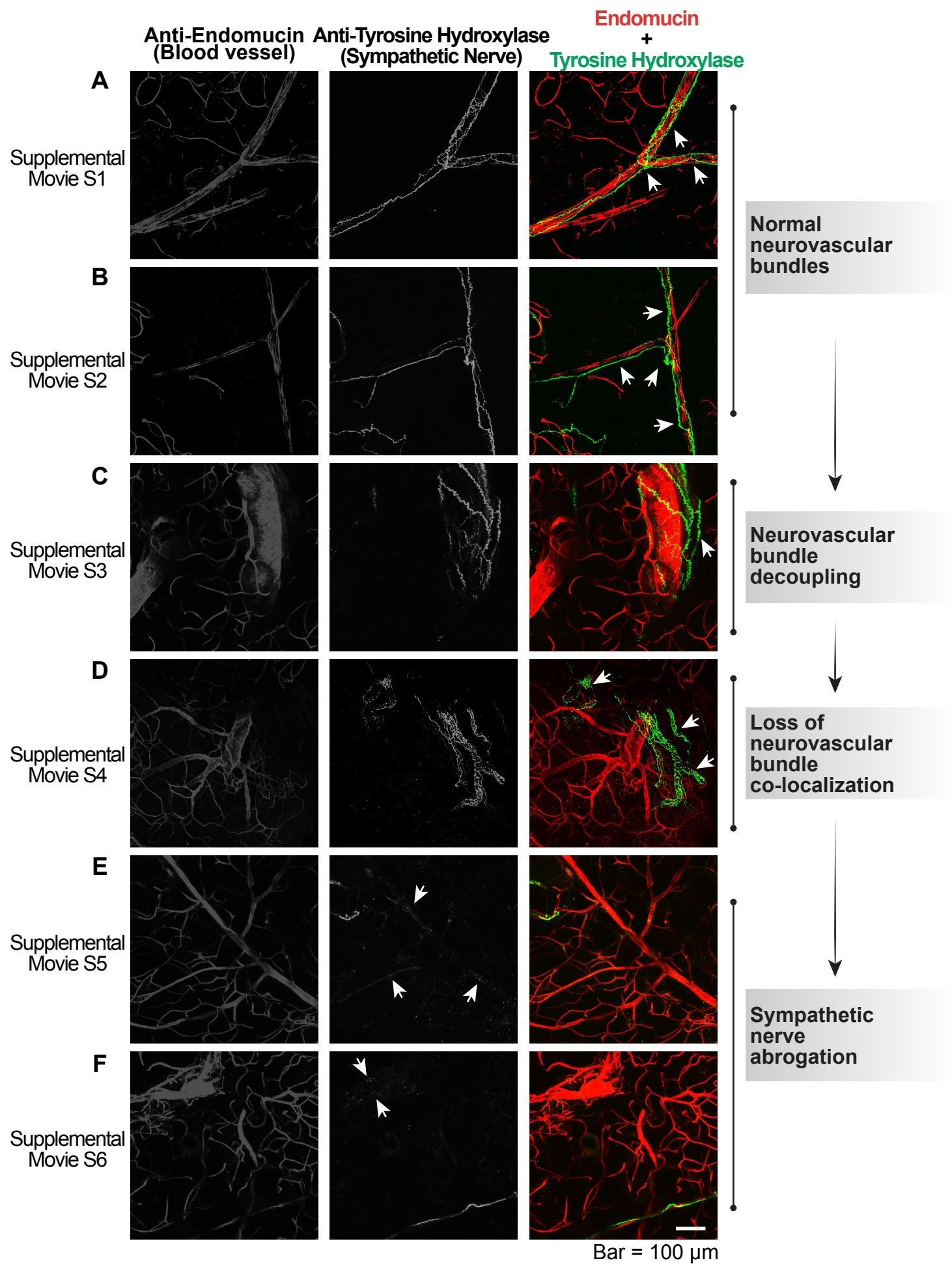

Supplement: Supplemental Figure S2: Representative images showing progressive pathological remodeling of neurovascular bundles in the Alzheimer’s disease model. [file mmc8.pdf]
